# Anti-Factor Xa Activity of Apixaban in Extremely Low Body Weight

**DOI:** 10.3390/jcm14155238

**Published:** 2025-07-24

**Authors:** Wanwarang Wongcharoen, Amarase Pamarapa, Siriluck Gunaparn, Arintaya Phrommintikul

**Affiliations:** Division of Cardiology, Department of Internal Medicine, Faculty of Medicine, Chiang Mai University, Chiang Mai 50200, Thailand; bwanwarang@yahoo.com (W.W.); amakawa.mc@gmail.com (A.P.); siriluck.g@cmu.ac.th (S.G.)

**Keywords:** atrial fibrillation, direct factor Xa inhibitor, coagulation factor Xa, body weight

## Abstract

**Background:** Direct oral anticoagulants (DOACs) are generally preferred over warfarin for preventing arterial and venous thromboembolism. However, the efficacy and safety of DOACs in patients with extremely low body weight (BW) are uncertain. This study investigates anti-factor Xa (anti-FXa) activity of apixaban and compares it between patients with normal BW (>50 kg) and underweight (≤50 kg). **Methods:** We enrolled 150 patients on branded generic apixaban (Apixan^TM^) for atrial fibrillation (AF), venous thromboembolism, and intracardiac thrombus. Anti-FXa activity of apixaban was measured at peak concentration (C_peak_) and trough concentration (C_trough_) after at least one week of therapy. **Results:** Mean age was 64.0 ± 12.7 years, with 53.3% being male. Mean BW was 61.3 ± 15.3 kg. Of the 150 patients, 132 (88%) had AF, and 43 (28.7%) had low BW. Overall, 87.3% and 84.7% of patients had C_trough_ and C_peak_ within the expected range. Underweight patients had significantly higher mean C_trough_ and C_peak_ than normal BW patients. A higher proportion of low-BW patients exceeded the expected C_peak_ range compared to normal-BW patients (25.6% vs. 3.7%, *p* < 0.001). Low BW was the only independent predictor of exceeding C_peak_ specified range (adjusted OR 4.87, 95% CI 1.31–18.15, *p* = 0.018). **Conclusions:** Most patients maintained apixaban levels within expected ranges, but those with low BW were more likely to exceed the specified range of C_peak_.

## 1. Introduction

Atrial fibrillation (AF) poses a significant global health challenge [1,2]. The incidence of AF is rising due to the aging population, leading to complications that adversely affect patients’ quality of life, including a five-fold-increased risk of ischemic stroke [3,4,5]. Over the past decade, direct oral anticoagulants (DOACs) have revolutionized anticoagulation therapy for patients with AF and are now preferred over warfarin [6].

Apixaban, a direct, oral, reversible, and highly selective inhibitor of factor Xa (FXa), has been demonstrated to be superior to warfarin in preventing stroke and systemic embolism. It is associated with a lower incidence of bleeding and reduced mortality in most clinical settings [7]. Prior studies have demonstrated a connection between the use of DOACs in underweight individuals and a heightened risk of major bleeding and mortality from any cause [8,9]. Nevertheless, the Apixaban for Reduction in Stroke and Other Thromboembolic Events in Atrial Fibrillation (ARISTOTLE) trial included a small proportion of patients with a body weight (BW) of 50 kg or less [7].

As the Asian population generally has lower BW compared to their Western counterparts, the use of DOACs in this lower BW range requires special caution. Lower BW can result in a reduced volume of distribution and slower drug clearance, potentially leading to unintentional supratherapeutic levels [10]. A pharmacokinetic study of apixaban conducted in 54 healthy subjects demonstrated that individuals weighing ≤ 50 kg had approximately 27% higher maximum observed plasma concentration and area under the concentration–time curve for apixaban [11]. However, there are currently no specific dose adjustments recommended for these patients.

Consequently, we aimed to assess the anti-FXa activity of apixaban to compare the proportion of patients whose levels fall within the expected range as defined by the European Heart Rhythm Association (EHRA) guidelines [12] between patients with normal BW (>50 kg) and those with extremely low BW (≤50 kg). Additionally, we sought to identify factors associated with anti-FXa activity exceeding the specified range.

## 2. Methods

### 2.1. Study Design

This was a single-center, open-label, prospective cohort study conducted at Maharaj Nakorn Chiang Mai Hospital, a tertiary care university hospital in Chiang Mai, Thailand. Investigators affiliated with Chiang Mai University designed the trial, collected data, and performed all statistical analyses. The study protocol received approval on 28 March 2023 (approval number 107/2023) from the ethics committee of the Faculty of Medicine, Chiang Mai University. The research adhered to the ethical principles of the Declaration of Helsinki, including obtaining written informed consent from all participants. The study is registered with the Thai Clinical Trials Registry (TCTR) at https://www.thaiclinicaltrials.org/ with the identification number TCTR20230410004, date of registration 7 April 2023.

### 2.2. The Studied Population

Adults aged ≥20 years with indication (s) for an oral anticoagulant, including clinical AF or atrial flutter (with non-gender CHA_2_DS_2_-VASc score ≥ 1), intracardiac thrombus, and venous thromboembolism were eligible to participate in the trial. Exclusion criteria included significant mitral stenosis, mechanical heart valve recipients, an estimated glomerular filtration rate (eGFR) < 15 mL/min/1.73 m^2^, and advanced chronic liver disease (Child–Turcotte–Pugh B or greater). Additionally, pregnant and breastfeeding women were excluded due to insufficient data on the use of DOACs in these conditions.

### 2.3. Trial Procedures

After obtaining the informed consent, the enrolled patients received Apixan^TM^, a branded generic apixaban manufactured by Intas Pharmaceuticals LTD, Ahmedabad, India. Berlin Pharmaceutical Industry (Bangkok, Thailand) supplied a one-year supply of branded generic apixaban for each participant but was not involved in the trial design or results. Each participant was prescribed either 5 mg or 2.5 mg of branded generic apixaban twice daily, with dose adjustments made according to standard clinical guidelines. All patients receiving apixaban 2.5 mg met at least two of the three criteria of reduced dose. Data on patients’ demographics, medical history, and current medications were collected. Blood samples were taken at least 7 days after the first dose of apixaban. These samples were collected in 3.2% sodium citrate tubes at trough and peak timepoints. They were centrifuged at 2500× *g* for 15 min at room temperature within 30 min of collection. These samples included the trough anti-FXa level, measured 15 min before the next dose of apixaban, and the peak anti-FXa level, measured 2–4 h after dosing. Additionally, complete blood counts, renal function tests, and basic coagulation tests were performed. Medication adherence was monitored through meticulous pill counts and verification of the exact time each participant took apixaban the previous day. Any patient who did not adhere to the medication regimen was scheduled for laboratory testing at a later date. Follow-up visits were scheduled every 3–4 months at the outpatient clinic to assess for side effects, adverse events, or potential drug discontinuation. Anti-FXa activity was measured using the Biophen^TM^ Heparin Liquid Reagent Technology (LRT) kit, a chromogenic anti-FXa assay from Hyphen BioMed (Neuville-sur-Oise, France). The assay was analyzed with the Sysmex CS-2500 system (Siemens Healthineers, Erlangen, Germany) and converted to apixaban plasma concentration using a commercial apixaban calibrator with a concentration sensitivity ranging from 0 to 700 mcg/L. The chromogenic assay for apixaban-specific anti-FXa activity showed a linear correlation with direct plasma concentration measurements obtained through liquid chromatography–tandem mass spectrometry (LC-MS-MS) [13].

### 2.4. Trial Outcomes

The primary objective of the present study was to evaluate the anti-FXa activity of branded generic apixaban (Apixan^TM^) in clinical practice and to compare apixaban peak (C_peak_) and trough (C_trough_) concentrations between patients with extremely low BW (≤50 kg) and those with normal BW (>50 kg). Additionally, we aimed to assess the proportion of patients exceeding expected therapeutic ranges and identify factors associated with supratherapeutic concentrations.

According to previous studies, the expected plasma concentrations of apixaban 5 mg at trough and peak ranged from 41 to 230 mcg/L and 91 to 321 mcg/L, respectively. For apixaban 2.5 mg, the expected C_trough_ and C_peak_ ranged from 34 to 162 mcg/L and 69 to 221 mcg/L, respectively [12,13].

### 2.5. Statistical Analysis

Descriptive statistics were used to analyze baseline characteristics and the primary outcome. Categorical variables, such as gender, presence of co-morbidities, and indications for oral anticoagulant (OAC) therapy, were presented as frequencies and percentages. Continuous variables, such as age and baseline laboratory results, were presented as means ± standard deviation. CHA_2_DS_2_-VASc and HAS-BLED scores as well as trough and peak apixaban plasma levels were reported as medians with interquartile ranges (IQR), both overall and by specific groups. The correlation between clinical factors and C_peak_ levels exceeding the expected value was assessed using binary logistic regression, with both univariate and multivariate analyses. Results are presented as odds ratios (OR) with 95% confidence intervals (CI) and corresponding *p*-values. All analyses were carried out by SPSS version 29.0.2.0 (IBM Corporation, Armonk, NY, USA).

## 3. Results

### 3.1. The Study Population

**Figure 1:**
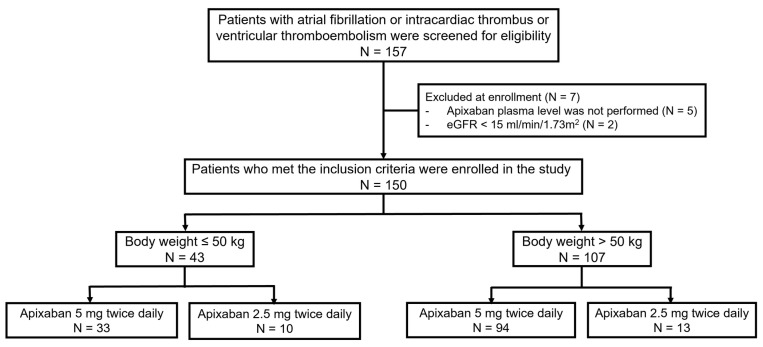
Consort flow diagram.

From May 2023 to January 2024, a total of 150 patients were enrolled in the trial (Figure 1). The majority of these patients (88.0%) required an OAC due to AF. The mean age of the entire cohort was 64 ± 13 years. The median BW was 58 kg (IQR 50–70 kg), with 43 patients (28.7%) weighing 50 kg or less. The mean age was significantly higher in the normal-weight group compared to the underweight group (69.71 ± 10.44 years vs. 61.74 ± 12.84 years, *p* < 0.001). Additionally, the proportion of males was higher in the normal-weight group compared to the underweight group (63.6% vs. 27.9%, *p* < 0.001). Creatinine clearance (CrCl) was significantly lower in the normal-weight group compared to underweight group (45.21 ± 21.60 mL/min vs. 70.34 ± 38.56 mL/min, *p* < 0.001). Overall, 84.7% of patients received the standard dose of apixaban, with no significant difference between the normal-weight and underweight groups. There were three (2%) patients receiving a non-dihydropyridine calcium channel blocker (non-DHP CCB), a moderate inhibitor of CYP3A4 and a weak inhibitor of P-glycoprotein. Detailed baseline characteristics of the study population are illustrated in Table 1.

### 3.2. The Study Outcomes

Overall, the median apixaban plasma levels were 101.60 (70.7–134.50) mcg/L and 185.60 (130.80–258.80) mcg/L for C_trough_ and C_peak_ in the standard-dose group and 72.30 (52.80–106.30) mcg/L and 122.30 (94.10–191.90) mcg/L for C_trough_ and C_peak_ in the reduced-dose group, as detailed in Table 2. The C_trough_ and C_peak_ were significantly higher in patients receiving the standard dose compared to those receiving the reduced dose of apixaban. Interestingly, we demonstrated that BW significantly influences plasma apixaban levels for the 5 mg dose but not for the 2.5 mg dose. Patients weighing ≤ 50 kg had significantly higher C_trough_ and C_peak_ of apixaban 5 mg compared to those weighing > 50 kg (*p* = 0.001 and *p* < 0.001, respectively). In contrast, for the 2.5 mg dose, there were no significant differences in C_trough_ and C_peak_ between the two body weight groups (*p* = 0.074 and *p* = 0.280, respectively).

**Figure 2:**
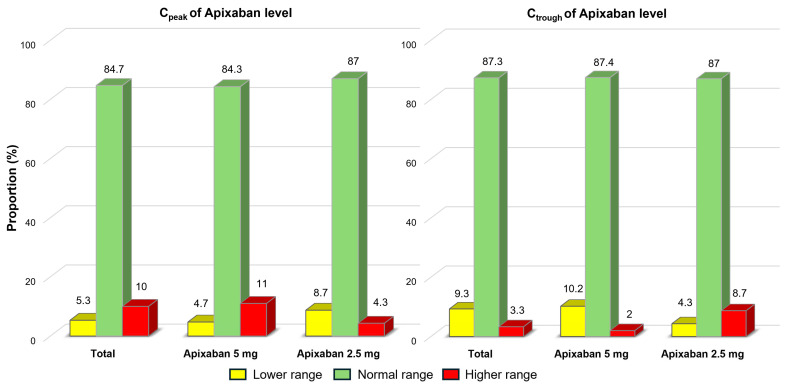
Proportions of patients with apixaban plasma levels within, below, and above the expected ranges in the entire cohort.

**Figure 3:**
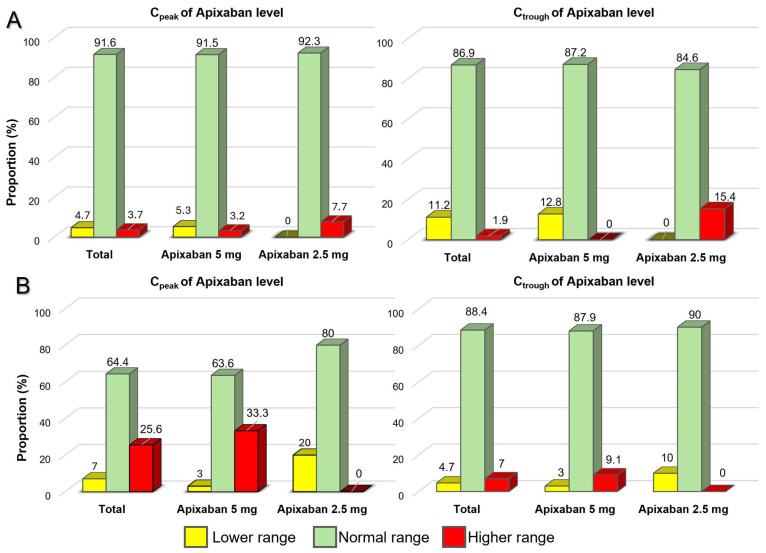
Proportions of patients with apixaban plasma levels within, below, and above the expected ranges between those with normal weight (**A**) and those weighing under 50 kg (**B**).

In the overall population, 87.3% of patients had their C_trough_ and 84.7% had their C_peak_ within the expected ranges, as shown in Figure 2. However, 3.3% of patients had their C_trough_ and 10.0% had their C_peak_ exceeded the expected ranges. Additionally, four patients (2.7%) had anti-FXa values that exceeded the expected range at both C_peak_ and C_trough_. When comparing patients with normal weight to those with low BW, it was observed that among individuals weighing more than 50 kg, 86.9% of patients had their C_trough_ and 91.6% had their C_peak_ within the expected ranges. In contrast, among patients weighing less than 50 kg, 88.4% of patients had their C_trough_ but only 67.4% had their C_peak_ within the expected ranges. Notably, 25.6% of patients with low BW had their C_peak_ exceeding the expected ranges. None of the underweight patients who received the reduced dose of apixaban had a C_peak_ that exceeded the expected range. However, 5.3% of these patients had a C_peak_ below the expected range. In contrast, 33.3% of the underweight patients who received the standard dose of apixaban had a C_peak_ that exceeded the expected range (Figure 3).

**Figure 4:**
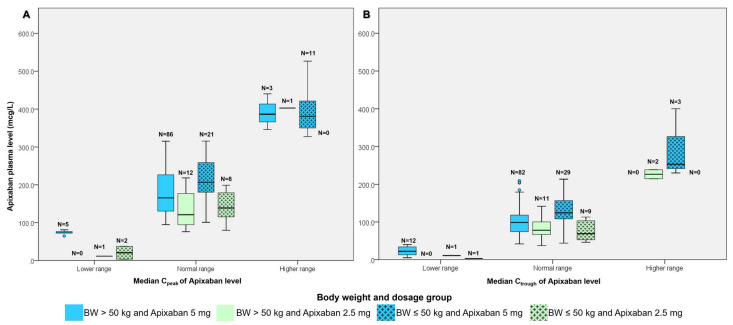
Median Cpeak levels (**A**) and median Ctrough levels (**B**) stratified by apixaban dose and body weight group.

Figure 4 illustrate the median C_peak_ and C_trough_ levels stratified by apixaban dose and BW group.

The univariate analysis showed that patients weighing ≤ 50 kg are more likely to have C_peak_ above the expected range (unadjusted OR 8.85, 95%CI 2.63–29.71, *p* < 0.001). While sex and CrCl may influence peak plasma levels of apixaban, these factors were not statistically significant in the multivariate analysis (Table 3). Other variables, such as age, medical history, or concurrent medications, did not show a correlation with C_peak_ levels exceeding the expected range. After adjusting for covariates in the multivariate analysis, low BW was the only independent predictor of exceeding peak concentrations of apixaban (adjusted OR 4.87, 95% CI 1.31–18.15, *p* = 0.018).

Apixaban was discontinued in eight patients (5.3%). Of these, two patients stopped the medication due to headaches, one due to the progression of chronic kidney disease requiring renal replacement therapy, and another due to the resolution of an intracardiac thrombus. The remaining discontinuations were attributed to personal reasons. During the median follow-up period of 11.0 ± 2.4 months, one patient experienced a traumatic intracerebral hemorrhage; however, both C_trough_ and C_peak_ levels were within the expected range for this patient. Additionally, two patients (1.3%) developed ischemic stroke. None of the patients experienced other significant bleeding during the follow-up period.

## 4. Discussion

In this single-center, prospective cohort study of plasma levels of a branded generic apixaban in 150 patients, we found that the majority of patients had their plasma levels within the expected range and that the drug is relatively well-tolerated. Our results are consistent with previous study on the anti-FXa activity of original apixaban in Thai patients [14]. The lower cost of generic DOACs can lead to their widespread use, improved adherence to treatment regimens, and better overall health outcomes. Our findings may provide reassurance to both healthcare providers and patients regarding the use of generic versions of DOACs.

Previous study has demonstrated that the use of DOAC in underweight patients was associated with the increased risk of major bleeding and all-cause mortality [8,9]. The patients with extremely low BW (≤50 kg) were underrepresented in the Apixaban for Reduction in Stroke and Other Thromboembolic Events in Atrial Fibrillation (ARISTOTLE) trial, where the median BW of participants was 82 kg [7], much higher than the median BW of 58 kg in our studied population.

Standard clinical practice guidelines for AF do not recommend routine plasma level testing for DOACs [12]. However, several studies have indicated the higher risk of bleeding in patients who have a plasma level of DOAC that exceeds the expected range [14,15,16]. Importantly, we demonstrated that the extremely low BW was an independent predictor of C_peak_ of apixaban exceeding the expected range. Our findings align with those from previous small studies. Stretton and colleagues reported that 50% of patients with a BW < 50 kg had unexpectedly high levels of DOACs, with the majority using apixaban. However, their study included only 10 patients weighing < 50 kg [17]. Similarly, Wolowiec and colleagues found comparable results, though their study included only seven underweight patients taking apixaban [18]. Several investigators have reported that apixaban has moderate lipophilic properties [19,20]. This increased susceptibility to unexpectedly high levels of apixaban in extremely low BW may be attributed to the reduced accumulation of the drug in adipose tissue and a propensity for higher drug concentrations in the blood.

While higher C_peak_ values in underweight patients are consistent with a reduced volume of distribution, the absence of a similar trend in the 2.5 mg subgroup observed in our study is noteworthy. This could suggest dose-dependent pharmacokinetics, though apixaban is generally described as having linear kinetics within therapeutic doses. Alternatively, variability in adherence, dosing intervals, or metabolism could contribute. Given the small size of the 2.5 mg subgroup, further studies are needed to clarify this observation.

Elevated C_peak_ and C_trough_ values can have important clinical implications for apixaban therapy. An increased C_peak_ may heighten the risk of bleeding due to greater anticoagulant effects [15]. A higher C_trough_ could indicate prolonged drug exposure, potentially leading to drug accumulation and an increased bleeding risk, though it may also enhance thrombotic protection by maintaining consistent anticoagulation [21]. When both C_peak_ and C_trough_ are elevated, overall drug exposure increases, amplifying bleeding risks without necessarily providing additional therapeutic benefits. These findings underscore the importance of therapeutic drug monitoring, especially in populations with low B, or other factors affecting drug metabolism, to optimize safety and efficacy.

Interestingly, no major bleeding events were observed despite 25.6% of low-BW patients having C_peak_ above the expected range. This may reflect that the reference range for C_peak_, as derived from pharmacokinetic studies, is conservative and may not always predict bleeding risk. Our finding aligns with results from the Japanese elderly AF patients (J-ELD AF) registry subanalysis, which found that low BW was not an independent predictor of increased stroke, bleeding, or mortality in elderly Japanese AF patients [22]. This consistency suggests that while pharmacokinetic differences exist, they may not necessarily translate to worse clinical outcomes with on-label apixaban dosing in that specific population. Alternatively, the relatively short observation period and the study’s design, which did not focus on bleeding outcomes, may have limited our ability to detect clinically significant events. Further longitudinal studies are needed to clarify the relationship between supratherapeutic C_peak_ and bleeding risk, particularly in underweight populations.

The National Institute for Health and Care Excellence (NICE) guidelines for venous thromboembolism recommend monitoring of DOAC level in patients who weigh less than 50 kg [23]. Similarly, the European Heart Rhythm Association (EHRA) guidelines suggest considering the plasma levels of DOACs in patients weighing under 50 kg [12]. Our results align with these recommendations, as extremely low BW was associated with a fivefold increased risk of apixaban levels exceeding the expected range in our study population.

Given our findings and existing guideline recommendations, we propose a practical clinical approach for managing underweight patients on apixaban: (1) initiate standard dose per indication; (2) in patients with BW ≤ 50 kg, especially those ≥80 years or with impaired renal function, consider measuring anti-FXa levels after steady state is achieved; (3) if C_peak_ exceeds expected range, assess for bleeding risk and consider dose adjustment or more frequent monitoring. This algorithm is intended to guide clinicians in the absence of formal dose-modification recommendations and should be validated in prospective studies.

Our study has several limitations. First, the sample size was relatively small. However, we believe that we enrolled a substantial proportion of patients with extremely low BW, a group often underrepresented in most DOAC studies. Nevertheless, the relatively small number of such patients may limit the statistical power to detect subtle differences in subgroup analyses, particularly in the reduced-dose group. Therefore, findings related to these subgroups should be interpreted with caution. Second, due to the very low incidence of stroke and bleeding events, we were unable to compare the efficacy and safety of apixaban between patients with normal BW and those with extremely low BW. Third, this study was conducted at a single center, which may limit the generalizability of the findings. Regional demographic factors such as age, body composition, and genetic background as well as adherence behaviors may influence apixaban levels and associated anti-FXa activity. Future multi-center studies including more diverse populations are warranted to confirm these results. Fourth, although we collected data on concurrent use of CYP3A4 and P-gp inhibitors, including non-DHP CCBs, the number of patients on these agents was too small to allow for a meaningful subgroup analysis. These patients were included in the overall analysis, which may have contributed to some variability in apixaban levels. This limitation should be considered when interpreting the results, and future studies should further evaluate the impact of drug–drug interactions on apixaban pharmacokinetics.

## 5. Conclusions

The majority of patients exhibited plasma levels within the expected range following administration of apixaban. Importantly, we demonstrated that individuals with extremely low BW were at an elevated risk of having peak anti-FXa activity levels that surpassed the expected range. Caution should be exercised when prescribing apixaban to individuals with extremely low BW.

## Figures and Tables

**Table 1:** Characteristics of the Patients at Baseline.

	Total (*n* = 150)	Body Weight ≤ 50 kg (*n* = 43)	Body Weight > 50 kg (*n* = 107)	*p*-Value *
Age (years)	64.03 ± 12.69	69.71 ± 10.44	61.74 ± 12.84	<0.001
Male (%)	80 (53.30%)	12 (27.90%)	68 (63.6%)	<0.001
Body mass index (kg/m^2^)	23.86 ± 4.74	19.24 ± 1.78	25.73 ± 4.26	<0.001
Hemoglobin (g/dL)	12.63 ± 2.06	11.57 ± 2.14	13.06 ± 1.89	<0.001
Serum creatinine (mg/dL)	1.13 ± 0.46	1.12 ± 0.44	1.14 ± 0.48	0.786
CrCl (ml/min)	63.13 ± 36.32	45.21 ± 21.60	70.34 ± 38.56	<0.001
Comorbidities (%)				
- Hypertension	67 (44.70%)	16 (37.20%)	51 (47.7%)	0.279
- Diabetes	40 (26.70%)	9 (20.90%)	31 (29.00%)	0.415
- Heart failure	59 (39.30%)	11 (25.60%)	48 (44.90%)	0.041
- CAD	38 (25.30%)	17 (39.50%)	21 (19.60%)	0.021
- CKD	19 (12.70%)	5 (11.60%)	14 (13.10%)	1.000
- Stroke	22 (14.70%)	4 (9.30%)	18 (16.80%)	0.312
OAC indication (%)				0.167
- Atrial fibrillation	132 (88.00%)	35 (81.40%)	97 (90.70%)	
- VTE	9 (6.00%)	5 (11.60%)	4 (3.70%)	
- Intracardiac thrombus	9 (6.00%)	3 (7.00%)	6 (5.60%)	
Medications (%)				
- Beta blocker	119 (79.30%)	32 (74.40%)	87 (81.30%)	0.376
- DHP CCB	29 (19.30%)	7 (16.30%)	22 (20.60%)	0.651
- Non-DHP CCB	3 (2.00%)	1 (2.30%)	2 (1.90%)	1.000
- Amiodarone	9 (6.00%)	2 (4.70%)	7 (6.50%)	1.000
- Flecainide	3 (2.00%)	1 (2.30%)	2 (1.90%)	1.000
- Statin	98 (65.30%)	25 (58.10%)	73 (68.20%)	0.259
Apixaban dose				0.130
Apixaban 5 mg twice daily	127 (84.70%)	33 (76.70%)	94 (87.90%)	
Apixaban 2.5 mg twice daily	23 (15.30%)	10 (23.30%)	13 (12.10%)	
CHA_2_DS_2_-VASc score Median (Q1, Q3)	3 (2, 4)	3 (2, 5)	3 (2, 4)	0.106
HAS-BLED score Median (Q1, Q3)	1 (1, 2)	2 (1, 3)	1 (1, 2)	0.536

* Compared between apixaban 5 mg twice daily and 2.5 mg twice daily. CAD = coronary artery disease; CKD = chronic kidney disease; CrCl = creatinine clearance; DHP CCB = dihydropyridine calcium channel blocker; OAC = oral anticoagulant; VTE = venous thromboembolism.

**Table 2:** Trough and peak apixaban plasma level at steady state.

Apixaban Dosage	Anti-Factor Xa Activity	Total	Body Weight ≤ 50 kg	Body Weight > 50 kg	*p*-Value *
Apixaban 5 mg *n* = 127	Trough apixaban plasma level (mcg/L) *	101.60 (70.7–134.50)	128.30 (104.9–165.00) (*n* = 33)	94.85 (65.38–113.75) (*n* = 94)	0.001
	Peak apixaban plasma level (mcg/L) *	185.60 (130.80–258.80)	258.80 (187.05–350.20) (*n* = 33)	163.45 (126.35–228.88) (*n* = 94)	<0.001
Apixaban 2.5 mg *n* = 23	Trough apixaban plasma level (mcg/L) *	72.30 (52.80–106.30)	63.75 (47.35–103.20) (*n* = 10)	79.10 (66.75–128.50) (*n* = 13)	0.074
	Peak apixaban plasma level (mcg/L) *	122.30 (94.10–191.90)	123.25 (69.18–172.23) (*n* = 10)	122.30 (94.55–195.50) (*n* = 13)	0.280

* Values are presented as median (Q1–Q3).

**Table 3:** Predictors of peak apixaban plasma level above expected range.

Predictors	Univariate Analysis Odds Ratio (95% CI)	*p*-Value	Multivariate Analysis Odds Ratio (95% CI)	*p*-Value
Female	5.31 (1.43–19.68)	0.013	3.18 (0.78–12.82)	0.104
Body weight ≤ 50 kg	8.85 (2.63–29.71)	<0.001	4.87 (1.31–18.15)	0.018
Creatinine clearance	0.97 (0.94–0.99)	0.033	0.98 (0.94–1.03)	0.563

## Data Availability

The deidentified participant data will be shared on a request-based basis. Please directly contact the corresponding author at arintaya.p@cmu.ac.th and the Faculty of Medicine, Chiang Mai University Ethics Committee to request data sharing.
